# Carbon Black: Kuempel et al. Respond

**DOI:** 10.1289/ehp.1103444R

**Published:** 2011-08-01

**Authors:** Eileen D. Kuempel, Paul A. Schulte, Thomas Sorahan, Jane Caldwell, Kurt Straif, Elizabeth Ward

**Affiliations:** National Institute for Occupational Safety and Health, Cincinnati, Ohio, E-mail: ekuempel@cdc.gov; Institute of Occupational and Environmental Medicine, University of Birmingham, Birmingham, United Kingdom; U.S. Environmental Protection Agency, Research Triangle Park, North Carolina; International Agency for Research on Cancer, Lyon, France; American Cancer Society, Inc., Atlanta, Georgia

We appreciate the comments and additional information from McCunney et al. We are pleased to learn of the new epidemiological studies that are under way in the U.S. and E.U. carbon black cohorts. These studies may provide the opportunity to fill some of the research gaps discussed in our review (Ward et al. 2010). As mentioned by McCunney et al. in their letter, we recommend the collection of particle size–specific and quantitative exposure data, and the recruitment of additional facilities (Ward et al.). Studies in animals have shown relationships between the particle surface area dose of poorly soluble particles (including carbon black) in the lungs and biomarkers of oxidative stress and inflammation in rats and mice (Elder et al. 2005; Sager and Castranova 2009; Stoeger et al. 2006) and lung tumors in rats (Driscoll 1996; Heinrich et al. 1995; Nikula et al. 1995). Although these relationships with particle surface area dose have not been reported in human studies, exposure to carbon black by particle mass has been associated with respiratory effects including lung function decrements in workers (Gardiner et al. 2001).

Concerning biomarkers of oxidative stress, we think the epidemiology studies under way may provide an opportunity to investigate and test hypotheses about possible biomarkers of exposure and response to carbon black. As we discussed in our paper (Ward et al. 2010), although oxidative stress has been invoked as a mechanism in the carcinogenicity of a number of agents (including particles such as carbon black), methodological challenges to the validation of oxidative stress biomarker assays remain. To facilitate this process, guidelines have been developed to standardize the collection and measurement of oxidative stress biomarkers in humans (American Thoracic Society 1999; Horváth et al. 2005).

We look forward to further reports from the carbon black mortality studies, including exposure–response analyses, which could help fill important occupational health research gaps. Well-conducted epidemiologic studies will be particularly critical to inform carcinogen classification and risk assessment processes.
